# Epidemiology of respiratory viral infections in people with acute respiratory tract infections in Africa: the VARIAFRICA systematic review and meta-analysis protocol

**DOI:** 10.1186/s13643-019-1037-1

**Published:** 2019-05-20

**Authors:** Sebastien Kenmoe, Jean Joel Bigna, Abdou Fatawou Modiyinji, Fredy Brice N. Simo, Richard Njouom

**Affiliations:** 1 Department of Virology, Centre Pasteur of Cameroon, 451 Rue 2005, P.O. Box 1274, Yaoundé, Cameroon; 2 Department of Epidemiology and Public Health, Centre Pasteur of Cameroon, 451 Rue 2005, P.O. Box 1274, Yaoundé, Cameroon; 3 School of Public Health, Faculty of Medicine, University of Paris Sud,, 63 Rue Gabriel Péri, 94270 Le Kremlin-Bicêtre, France; 4 Department of Biology and Animal Physiology, Faculty of Sciences, University of Yaoundé 1, P.O. Box 337, Yaoundé, Cameroon; 5 Department of Biochemistry, Faculty of Sciences, University of Yaoundé 1, P.O. Box 337, Yaoundé, Cameroon

**Keywords:** Epidemiology, Respiratory viruses, Acute respiratory tract infections, Africa

## Abstract

**Introduction:**

Better characterisation of the epidemiological data on respiratory viral infections among people with acute respiratory tract infection (ARTI) can help to implement efficient strategies to curb the burden of ARTI in Africa. We will conduct a systematic review and meta-analysis to determine the prevalence and factors associated with respiratory viral infection in people of all ages with ARTI residing in Africa.

**Methods:**

This work will include cross-sectional studies published between January 1, 2000 and December 31, 2017, without any language restriction, on populations residing in African countries. We will consider studies that reported the prevalence of respiratory viruses in people with ARTI confirmed by a polymerase chain reaction technique. We will be searching PubMed, Embase, African Journals Online, Web of Science, and Global Index Medicus. The selection of relevant studies, extraction of data, and evaluation of the quality of the articles will be carried out independently by two review authors, and the discrepancies will be resolved by consensus or intervention of a third author. The heterogeneity of the studies will be assessed using the *χ*^*2*^ test on Cochrane’s *Q* statistic. Publication bias will be assessed by the Egger test. Studies will be pooled using a random-effect meta-analysis model. Results will be presented by age group and sub-region of Africa. Using meta-regression models, we will identify factors associated with viral infections in people with ARTI.

**Discussion:**

This systematic review and meta-analysis is based on published data and therefore does not require ethical approval. This work will serve as a basis for the development of strategies for prevention and control ARTI in Africa and will also serve to identify data gaps and guide future investigations. The final report will be published in peer-reviewed journals as a scientific article and presented in workshops, conferences, and scientific conferences.

**Systematic review registration:**

PROSPERO, CRD42018088261.

**Electronic supplementary material:**

The online version of this article (10.1186/s13643-019-1037-1) contains supplementary material, which is available to authorized users.

## Background

Data on people of all ages from a meta-analysis show that about 3 million deaths were caused by acute lower respiratory infections in 2015 [1]. The burden of these infections is essentially supported by Sub-Saharan Africa which recorded about a quarter of all deaths (0.7 million death) [1]. Several viruses have been identified as etiologic agents responsible for ARTI. The clinically most important are classified into 6 families which include Paramyxoviridae (human respirovirus 1 and 3, human rubulavirus 2 and 4 previously named Parainfluenza virus 1–4), Pneumoviridae (respiratory syncytial virus A and B and human metapneumovirus A and B), Picornaviridae (enterovirus A to J and rhinovirus A to C), Coronaviridae (human coronavirus: HCoV-229E, HCoV-OC43, HCoV-NL63, HCoV-HKU1, SARS-CoV and HCoV-EMC), Orthomyxoviridae (Influenza A, B, and C), and Adenoviridae (human adenovirus A to G) [2]. The ecology of Africa can give to this continent a specific epidemiological profile for ARTI. To date and to the best of our knowledge, one systematic review of data published between 2000 and 2015 on the prevalence of respiratory pathogens only in children under 5 living in Africa has been reported [3]. This systematic review considered only data from sub-Saharan Africa did not perform a meta-analysis and quality assessment of the included articles. In the present systematic review with meta-analysis, we will include population regardless of age and from the entire African continent including Northern Africa. We will therefore able to compare the epidemiology of viral aetiologies of acute respiratory tract infections among age groups and among geographical regions of the continent.

## Objectives

To address this gap of knowledge, we will conduct a systematic review and meta-analysis of the Viral Aetiology of Acute Respiratory Infections in Africa (VARIAFRICA) on people of all age groups to describe the epidemiology of respiratory viral infections (human respiratory syncytial virus, metapneumovirus, influenza virus, rhinovirus, adenovirus, bocavirus, parainfluenzavirus, coronavirus, and enterovirus). Specifically, we will report prevalence and factors associated with high prevalence. This work is done to provide accurate data to guide health decision makers and to identify information gaps to guide future research on the burden of viral infections in ARTI Africa.

## Method/design

### Design

This systematic review and meta-analysis will be conducted in accordance with Centre for Reviews and Dissemination guidelines [4]. This protocol has been reported following the recommendations for preferred reporting items for systematic reviews and meta-analyses for protocol (Additional file 1: Table S1) [5]. This review has been registered with PROSPERO, CRD42018088261.

### Eligibility criteria

#### Inclusion criteria

This study will include the following:

1. Types of studies: cross-sectional studies.
2. Participants: adults and children with ARTI residing in Africa continent.
3. Outcome: studies reporting the prevalence of human respiratory syncytial virus, metapneumovirus, influenza virus, rhinovirus, adenovirus, bocavirus, parainfluenzavirus, coronavirus, and enterovirus (or enough data to compute this estimate). The diagnosis of viruses has to be done with polymerase chain reaction techniques.
4. Period: studies published between January 1, 2000 and December 31, 2017.

#### Exclusion criteria

1. Studies conducted during an outbreak period.
2. Case reports, letters, commentaries, and editorials.
3. Studies with imported cases of ARTI.
4. In the case of duplicates, only the study with the largest sample size will be considered.
5. Studies whose full texts are not available or the data are not complete even after the request to the authors.

### Search strategy for identifying relevant studies

A comprehensive search PubMed, Excerpta Medica Database, African Journals Online, Web of Science, and Global Index Medicus will be conducted to identify all relevant articles published on ARTI in people living in Africa from January 1, 2000 to October 31, 2017, without any language restriction. The search strategy in PubMed is presented in Additional file 2: Table S2. This search strategy will be adapted to fit other databases. The references of the eligible articles and relevant reviews will be manually searched to identify additional studies.

### Selection of studies for inclusion in the review

Duplicates will be removed using EndNote 7. Two reviews authors will independently assess the titles and/or abstracts of eligible articles according to the inclusion and exclusion criteria using Rayyan Online application. Studies in a language different from English, French, or Spanish will be translated using Google Translate and considered for eligibility. Two review authors will independently evaluate the full text of the selected records. Discrepancies will be resolved by consensus or will involve a third review author as an arbitrator. The agreement between the two authors will be estimated by Cohen’s kappa coefficient [6].

### Risk of bias assessment

The risk of bias tool developed for prevalence studies by Hoy et al. will be used to assess the risk of bias of finally included studies [7]. The defined items will be scored with 0 for ‘no’ and 1 for ‘yes.’ The total score of each article will be calculated by the sum of its items. The studies will be ranked according to their total score as low risk, moderate risk, and high risk for scores of 8–10, 5–7, and 0–4, respectively. Two review authors will assess the risk of bias and disagreements will be solved through a consensus or by arbitration of a third review author.

### Data extraction and management

A preconceived and tested questionnaire will be used to collect data (name of the first author, year of publication, design of the study, location, sampling method, period of study, timing of sample collection, number of viruses searched, country, city, latitude, longitude, altitude, clinical presentation, number of patients tested for respiratory viral infections, number of patients infected with viruses, diagnostic technique used, and male proportion).The countries will be grouped into regions according to the United Nations Statistics Division. ARTI will be classified as severe infections (severe acute respiratory infections, acute lower respiratory infections, bronchitis, bronchiolitis and pneumonia) and benign infections (upper respiratory tract infections and influenza-like illness). Two review authors will independently extract data. Discrepancies between the two review authors will be resolved by consensus or by a third review author if necessary. Authors of included studies will be contacted to request information in case of missing data.

### Data synthesis and analysis

Data will be analysed using the *‘meta’* packages of the statistical software R (version 3.5.1, The R Foundation for statistical computing, Vienna, Austria). Unadjusted prevalence will be recalculated based on the information of crude numerators and denominators provided by individual studies. Prevalence will be reported with their 95% confidence interval and prediction interval. To keep the effect of studies with extremely small or extremely large prevalence estimates on the overall estimate to a minimum, the variance of the study -specific prevalence will be stabilised with the Freeman-Tukey double arcsine transformation before pooling the data with the random-effects meta-analysis model [8]. Egger’s test will serve to assess the presence of publication bias [9]. A *p* value < 0.10 on Egger test will be considered indicative of statistically significant publication bias. Heterogeneity will be evaluated by the *χ*^*2*^ test on Cochrane’s *Q* statistic [10], which will be quantified by *H* and *I*^2^ values. The *I*^2^ statistic estimates the percentage of total variation across studies due to true between-study differences rather than chance. In general, *I*^2^ values greater than 60–70% indicate the presence of substantial heterogeneity [11]. Subgroup analyses will be performed for the following subgroups: age groups (0–5 years versus > 5 years), population (children [≤ 15 years] versus adults), clinical presentation (severe versus benign forms), and UNSD African Regions. Univariable meta-regression will be used to test for an effect of study and participants’ characteristics (year of publication, seasonality, number of screened viruses, clinical presentation, age groups, population, UNSD of regions, absolute latitude [distance to equator], latitude, longitude, and altitude). Following crude overall prevalence, we will conduct two sensitivity analyses to assess the robustness of our findings. The first one will include only studies with low risk of bias and the second only studies reporting data of a full year(s) period (complete season(s)).

### Potential amendments

We do not plan to make any changes to this protocol. However, if substantial changes occur during the review process, they will be reported in the published results.

### Ethics and dissemination

This work is based on published data and therefore does not require an ethical approval. This systematic review and meta-analysis should serve as a basis for designing preventive and control strategies for ARTI in Africa, and serve as a guide for future research based on the identification of the remaining gaps. The results of this study, in the form of scientific article, will be published in a peer-reviewed journal. These results will also be presented at conferences and submitted to relevant public health authorities. We also plan to update the review in the future to monitor changes and guide solutions for health services and policies.

## Discussion

The overall objective of this systematic review is to inform public health stakeholders about the burden of respiratory viral infections in people with ARTI in Africa and to provide information that can support actions to optimise their decisions. We hope that this work will serve as a sink for the consideration of this major public health concern and to identify the priorities for future research in the field. To the best of our knowledge, this will be the first systematic review and meta-analysis that will report the prevalence of viral aetiologies of ARTI in Africa including children and adults. There would be some heterogeneity in the definition of cases of ARTI according to studies.

## Additional files

Additional file 1: PRISMA-P (Preferred Reporting Items for Systematic review and Meta-Analysis Protocols) 2015 checklist: recommended items to address in a systematic review protocol*. (PDF 225 kb)

Additional file 2: **Table S1.** Search strategy in PubMed. (PDF 122 kb)

## Abbreviations

ARTI: Acute respiratory tract infection
VARIAFRICA: Viral Aetiology of Acute Respiratory Infections in Africa.
