# Supplementary material for: Epidemiology of respiratory viral infections in people with acute respiratory tract infections in Africa: the VARIAFRICA systematic review and meta-analysis protocol
Source: Syst Rev. 2019 May 20;8:122. doi: 10.1186/s13643-019-1037-1 (PMC6528219; doi:10.1186/s13643-019-1037-1)
Supplement: Supplementary file 2 — Table S1. Search strategy in PubMed. (PDF 122 kb) [file 13643_2019_1037_MOESM2_ESM.pdf]

Supplemental Table 1. Search strategy in PubMed

Search	Search terms
#1	“HRSV” OR “RSV” OR “human respiratory syncytial virus” OR “respiratory syncytial virus” OR “HRSV A” OR “HRSV B”
#2	“HMPV” OR “MPV” OR “human metapneumovirus” OR “metapneumovirus” OR “HMPV A” OR “HMPV B”
#3	“HAdV” OR “AdV” OR “Adenovirus” OR “Adenovirus Infect*” OR “Human adenovirus” OR “human adenovirus infection” OR “HADV A” OR “HADV B” OR “HADV C” OR “HADV D” OR “HADV E” OR “HADV F” OR “HADV G”
#4	“HBoV” OR “BoV” OR “Bocavirus” OR “Bocavirus Infections, Human” OR “Human Bocavirus” OR “Bocavirus Infect*”
#5	“HCoV” OR “CoV” OR “Coronavirus” OR “Coronavirus Infections, Human” OR “Human Coronavirus” OR “229E” OR “OC43” OR “NL63” OR “HKU1” OR “HCoV 229E” OR “HCoV OC43” OR “HCoV NL63” OR “HCoV HKU1”
#6	“HPIV” OR “PIV” OR “Parainfluenzavirus” OR “Parainfluenzavirus Infect*” OR “Human Parainfluenzavirus Infect*” OR “Human Parainfluenzavirus” OR “PIV1” OR “PIV2” OR “PIV3” OR “PIV4” OR “HPIV1” OR “HPIV2” OR “HPIV3” OR “HPIV4” OR “Paramyxoviridae” OR “Paramyxoviridae virus” OR “Paramyxoviridae infection”
#7	“HEV” OR “EV” OR “Enterovirus” OR “Enterovirus Infections, Human” OR “Human Enterovirus”
#8	“HRV” OR “Rhinovirus” OR “Rhinovirus Infect*” OR “Human Rhinovirus” OR “RVA” OR “RVB” OR “RVC”
#9	“Influenza virus” OR “Human Influenza” OR “Influenza” OR “Human Flu” OR “Influenza A virus” OR “Influenza B virus” OR “Influenza C virus” Or “Grippe”
#10	“lower respiratory tract infect*” OR “LRTI” OR “acute lower respiratory infect*” OR “ALRI” OR “pneumonia” OR “community acquired pneumonia” OR “bronchiolitis” OR “severe acute respiratory infect*” OR “severe acute respiratory illness” OR “ILI” OR “Influenza Like Illness” OR “whooping cough” OR “bronchopneumonia” OR “pleurisy” OR “pleuropneumonia” OR “bronchitis” OR “respiratory infect*” OR “upper respiratory tract infect*” OR “upper respiratory infect*”
#11	Africa* OR Algeria OR Angola OR Benin OR Botswana OR "Burkina Faso" OR Burundi OR Cameroon OR "Canary Islands" OR "Cape Verde" OR "Central African Republic" OR Chad OR Comoros OR Congo OR "Democratic Republic of Congo" OR Djibouti OR Egypt OR "Equatorial Guinea" OR Eritrea OR Ethiopia OR Gabon OR Gambia OR Ghana OR Guinea OR "Guinea Bissau" OR "Ivory Coast" OR "Cote d'Ivoire" OR Jamahiriya OR Kenya OR Lesotho OR Liberia OR Libya OR Madagascar OR Malawi OR Mali OR Mauritania OR Mauritius OR Mayotte OR Morocco OR Mozambique OR Namibia OR Niger OR Nigeria OR Principe OR Reunion OR Rwanda OR "Sao Tome" OR Senegal OR Seychelles OR "Sierra Leone" OR Somalia OR "South Africa" OR “South Sudan” OR "St Helena" OR Sudan OR Swaziland OR Tanzania OR Togo OR Tunisia OR Uganda OR "Western Sahara" OR Zaire OR Zambia OR Zimbabwe OR "Central Africa" OR "Central African" OR "West Africa" OR "West African" OR "Western Africa" OR "Western African" OR "East Africa" OR "East African" OR "Eastern Africa" OR "Eastern African" OR "North Africa" OR "North African" OR "Northern Africa" OR "Northern African" OR "South African" OR "Southern Africa" OR "Southern African" OR "sub Saharan Africa" OR "sub Saharan African" OR "sub Saharan Africa" OR "sub Saharan African”
#12	#1 OR #2 OR #3 OR #4 OR #5 OR #6 OR #7 OR #8 OR #9
#13	#10 AND #11 AND #12
#14	("2000/01/01"[Date - Publication] : "3000"[Date - Publication])
#15	#13 AND #14
